# Sense-antisense gene pairs: sequence, transcription, and structure are not conserved between human and mouse

**DOI:** 10.3389/fgene.2013.00183

**Published:** 2013-09-26

**Authors:** Emily J. Wood, Kwanrutai Chin-Inmanu, Hui Jia, Leonard Lipovich

**Affiliations:** ^1^Center for Molecular Medicine and Genetics, Wayne State UniversityDetroit, MI, USA; ^2^Bioinformatics Program, School of Information Technology and School of Bioresources and Technology, King Mongkut's University of Technology Thonburi (KMUTT)Bangkok, Thailand

**Keywords:** sense-antisense, transcriptome, long non-coding RNA (lncRNA), expressed sequence tags (ESTs), evolution, complex loci, bidirectional promoters

## Abstract

Previous efforts to characterize conservation between the human and mouse genomes focused largely on sequence comparisons. These studies are inherently limited because they don't account for gene structure differences, which may exist despite genomic sequence conservation. Recent high-throughput transcriptome studies have revealed widespread and extensive overlaps between genes, and transcripts, encoded on both strands of the genomic sequence. This overlapping gene organization, which produces sense-antisense (SAS) gene pairs, is capable of effecting regulatory cascades through established mechanisms. We present an evolutionary conservation assessment of SAS pairs, on three levels: genomic, transcriptomic, and structural. From a genome-wide dataset of human SAS pairs, we first identified orthologous loci in the mouse genome, then assessed their transcription in the mouse, and finally compared the genomic structures of SAS pairs expressed in both species. We found that approximately half of human SAS loci have single orthologous locations in the mouse genome; however, only half of those orthologous locations have SAS transcriptional activity in the mouse. This suggests that high human-mouse gene conservation overlooks widespread distinctions in SAS pair incidence and expression. We compared gene structures at orthologous SAS loci, finding frequent differences in gene structure between human and orthologous mouse SAS pair members. Our categorization of human SAS pairs with respect to mouse conservation of expression as well as structure points to limitations of mouse models. Gene structure differences, including at SAS loci, may account for some of the phenotypic distinctions between primates and rodents. Genes in non-conserved SAS pairs may contribute to evolutionary lineage-specific regulatory outcomes.

## Introduction

A sense-antisense (SAS) gene pair is defined as two genes that reside on opposite genomic strands within the same locus and share exonic sequence overlap. Until recently, the genome was thought to be organized into discrete transcriptional units (TUs). This assumption contrasts with the unanticipated complexity of gene structure revealed by large-scale transcriptome sequencing projects (Derrien et al., 2012). The number of complex loci, in which TUs are joined at the sequence level by SAS overlap or bi-directional promoters (when transcription start sites fall within 1 Kb of each other) in the human and mouse genomes is significantly higher than expected by chance: 25% of all transcripts in both species may have SAS partners and up to 10% of genes in the human genome participate in bi-directional promoters (Engström et al., 2006). Thousands of SAS pairs have been identified in human and mouse (Li et al., 2008; Grinchuk et al., 2010), and hundreds in numerous model organisms, with more expected due to advancing technology (Babak et al., 2007). *D. melanogaster* and *C. elegans* genomes have abnormally high and low SAS pair content respectively; pair incidence is more uniform in vertebrates (Chen et al., 2005; Kutter et al., 2012). SAS pairs also occur in fungi (Prescott and Proudfoot, 2002; Hongay et al., 2006) and prokaryotes (Storz et al., 2005; Georg and Hess, 2011).

**Figure 1:**
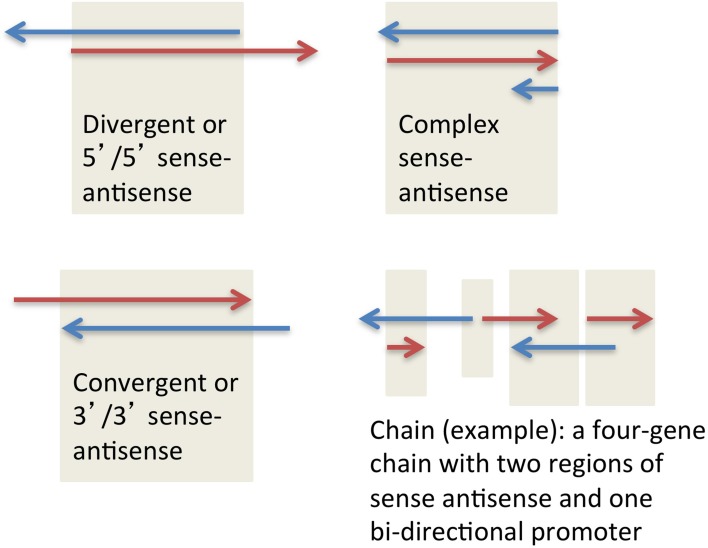
**Three major types of sense-antisense pairs, and one possible type of a gene chain**.

SAS pairs in all species analyzed to date contain both protein-coding genes and non-coding RNA genes, most often one coding and one non-coding in each pair. SAS pairs can be structurally classified as divergent, convergent, and complex (Figure 1). These configurations proportionally make up 55, 20, and 25% respectively of SAS pairs in the human genome (Grinchuk et al., 2010). The complex category includes nested and embedded pairs as well as any additional scenarios other than simple overlaps of sense and antisense genes at their 5′ ends (divergent) or 3′ ends (convergent).

SAS regulation and small-RNA pathways are mechanistically distinct. Small regulatory RNAs include microRNAs (miRNAs), each of which regulates multiple mRNAs encoded outside of its own locus, and endogenous small-interfering RNAs (siRNA) (Smalheiser, 2012). Their pathways utilize DICER and RISC, and are collectively described as RNA interference (RNAi). Unlike small RNAs, endogenous SAS long non-coding RNA (lncRNA) molecules originate from the same locus as the genes they regulate, are canonically processed (including a 5′ 7-methylguanylate cap, intron removal, and 3′ polyadenylation), and are usually transcribed by RNA-polymerase II (Lipovich et al., 2010). SAS overlaps can undergo RNA editing (Peters et al., 2003). Recent work has implicated small-RNA pathways in SAS-lncRNA regulation (Morris et al., 2008).

Despite reciprocal regulation at specific SAS loci where sense and SAS expression levels are inversely correlated, SAS expression analyses of such loci indicate widespread synergistic co-expression of sense and antisense transcripts (Yelin et al., 2003; Engström et al., 2006). Sense and antisense RNA levels originating from these loci vary concordantly upon a specific stimulus or after RNAi- or overexpression-induced perturbation of one of the two transcripts in the SAS pair (Katayama et al., 2005; Engström et al., 2006). Microarray evidence points to dynamic and cell-specific regulatory patterns of SAS pairs (Oeder et al., 2007; Numata et al., 2009).

Endogenous antisense transcription has a plethora of documented mechanisms and functions. Although we focus on cis-encoded antisense transcripts (arising from the same locus as their sense counterparts), in-trans regulation of mRNAs by antisense lncRNAs is also important, particularly in mRNA degradation (Gong and Maquat, 2011). The transcription factors β-Catenin and TCF4 induce endogenous antisense transcription in the locus encoding E2F4, another transcription factor, resulting in an antisense-mediated reduction of E2F4 protein level with an accompanying decrease in E2F4 binding to target promoters (Yochum et al., 2007). In the thymidine synthase (TS) locus, specific splice isoforms of the SAS RNA (rTS) are necessary and sufficient for TS mRNA down regulation (Izant and Weintraub, 1984; Chu and Dolnick, 2002). SAS transcription exerts a key function in mammalian X-chromosome inactivation: the non-coding RNA Tsix, activated by pluripotency transcription factors, serves as a cis-antisense repressor of another non-coding RNA, Xist, which in turn silences much of the inactive X-chromosome through recruiting the histone-modifying repressor complex PRC2 (Jeon et al., 2012). In the mouse Crx locus, the cis-antisense transcript reduces sense-encoded protein levels (Alfano et al., 2005; Hsiau et al., 2007), suppressing a transcription factor.

Post-transcriptional positive regulation of sense protein-coding genes by antisense lncRNAs has been experimentally verified at the BACE1 locus where the SAS transcript BACE1-AS masks a miRNA binding site in BACE1 mRNA, stabilizing the transcript (Faghihi et al., 2008). Summarily, as a result of the functional diversity and mechanistic heterogeneity of endogenous antisense transcripts, which can occur both in the nucleus and in the cytoplasm, antisense transcription can be both a positive and a negative regulator of gene expression (Katayama et al., 2005; Lipovich et al., 2012). SAS transcription regulates alternative splicing (Salato et al., 2010). Cytosolic decapping of antisense lncRNAs may activate sense mRNA partners (Geisler et al., 2012).

Despite the multiplicity of antisense mechanisms and the importance of antisense functions, evolutionary conservation of SAS pairs has been reported as low, even between closely related mammals (Galante et al., 2007). The earliest, highly conservative analyses of human and mouse SAS pairs already alluded to the possibility that antisense overlaps specific to restricted evolutionary lineages arose after the mammalian radiation (Shendure and Church, 2002). Human overlapping, including SAS, genes are characterized by an overrepresentation of those which lack homologs in other vertebrate genomes (Makalowska et al., 2005), an idea in agreement with the overprinting hypothesis (Keese and Gibbs, 1992) and consistent with non-adaptive exaptation phenomena through which genomic sequences can acquire new functions (Brosius and Gould, 1992). At antisense loci, the functional requirement for antisense may be sequence-independent (Carninci and Hayashizaki, 2007). However, no systematic tests of the hypothesis that SAS pair members arose in recent evolution from lineage-specific or previously non-genic sequences have been performed to date.

Previous work utilized sequence alignments to assess the level of conservation between mouse and human at SAS loci. The FANTOM3 Consortium observed that less than 20% of over 5000 SAS pairs analyzed displayed evidence of conservation at orthologous SAS overlap regions (Engström et al., 2006). A similar analysis limited to known genes found only a 6.6% conservation rate of SAS pairs (Numata et al., 2007). While low interspecies conservation of sequence and genomic structures in SAS pairs is evident, there has been a paucity of studies addressing the reasons why SAS loci are poorly conserved. In the MINK/CHRNE locus, where a convergent SAS overlap exists in some mammals but convergent gene orientation without overlap is evident in others, the gene structure difference has been traced to generation and destruction of canonical polyadenylation signals through indels and single-base substitutions after the mammalian radiation (Dan et al., 2002). These changes, which lead to interspecies differences in gene 3′-boundary locations at this locus, dictate the possibility of a SAS overlap or lack thereof in each species. In rodents, a low empirical rate of de-novo exon generation from non-transposon lineage-specific sequences has been demonstrated (Wang et al., 2005), but mechanisms of *de-novo* exon generation have only recently begun to be elucidated (Carvunis et al., 2012).

The goal of our study is to assess the genomic sequence, gene structure, and expression conservation of SAS pairs between human and mouse; and to test whether our earlier findings (Lipovich et al., 2006) of SAS non-conservation are reflective of a genome wide trend. We hypothesized that a comparison of orthologous complex loci between mouse and human will identify locations where gene structure and transcriptional activity differences, capable of exerting lineage-specific cis-regulatory outcomes, have arisen after the divergence of rodents and primates.

## Materials and methods

### Construction of the human SAS dataset

We inferred a set of human TUs from Genbank cDNA and EST evidence, and used that set to globally identify putative SAS pairs, using our previously described computational pipeline (Lipovich and King, 2006). Reference transcripts (one cDNA or EST per each gene in each SAS pair) were selected by manual curation to reflect the most frequently used sites of transcription initiation, splicing, and polyadenylation for each TU. Reference transcripts were mapped to the human hg19 assembly by parsing ref_all, all_mrna and all_est files from the UCSC Genome Database (Kent et al., 2002) to retrieve mappings based on Genbank accession numbers. Gene names were assigned to SAS pair members, where possible, by assessing the orientation and positional relationship of reference transcripts relative to UCSC KnownGenes and RefSeq track entries sharing the locus.

Additionally, we mapped four publicly available human SAS datasets from other groups [(Shendure and Church, 2002; Yelin et al., 2003; Veeramachaneni et al., 2004), and (Chen et al., 2005)] to hg19, using BLAT (Kent, 2002). We then eliminated intra-dataset redundancy of the five datasets, resulting in a 6718-pair interim dataset. Finally, through extensive manual annotation which encompassed a review of each locus in the UCSC Genome Browser, we eliminated non-redundant pairs whose reference transcripts had ambiguous genomic mappings, exhibited orientation inconsistencies, and/or contained non-canonical splice sites and/or non-canonical polyadenylation signals, yielding the final 4511-pair dataset. This dataset was previously reported in Grinchuk et al. (2010), with minor differences in pair counts due to changes in UCSC Genome Database transcript-to-genome alignments.

### Interspecies genome coordinate conversions and identification of mouse genomic regions orthologous to human SAS pairs

**Figure 2:**
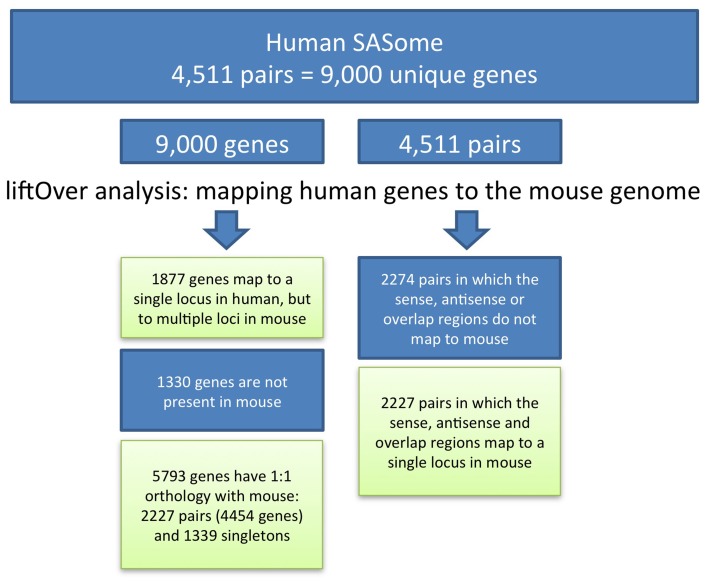
**Conservation of SAS gene pairs and their member genes between human and mouse**.

To convert genome positions of human SAS genes to the mouse genome assembly, we applied the UCSC LiftOver tool, which utilizes pre-computed BLASTZ pairwise alignments. Human SAS pair member genes, including genomic spans, individual exons, and SAS overlap regions, were aligned to the mouse mm9 assembly. The Linux LiftOver executable was obtained from www.soe.ucsc.edu/~kent/exe/linux/liftOver.gz and batch-executed using the command line:./*liftover input.bed over.chain output.bed error.bed*. BED files containing the columns chromosome, start, end, sequence ID were created for each gene. Using batch UCSC LiftOver we mapped members of each human SAS pair to mouse. We mapped each gene individually (gene-level LiftOver) and we also mapped the genomic span of both genes and the overlap (pair-level LiftOver) (Figure 2). The “multiple map” LiftOver option was used. Therefore, some human genome coordinate sets mapped to mouse at more than one location (because of duplications, non-orthologous homologs, or genome assembly problems). For each human gene, the LiftOver result was classified according to the number of matching positions in the mouse, as mapping to single, multiple, or no mouse locations. Any human query may or may not be conserved in mouse; any conserved query (a SAS gene pair) may be detectable at any location in mouse (i.e., either together as a pair at the same locus, or as two genes at different loci); and the conservation of any query, such as a single SAS pair member gene, in the interspecies alignment may be either at a single mouse genomic location or over multiple loci.

### Transcriptional activity analysis of mouse genomic regions orthologous to human SAS pairs

After identifying mouse regions putatively orthologous to human SAS gene pairs, we searched for biological evidence of transcription at these sites. First, we tested whether any mouse mRNA/cDNA sequences from Genbank mapped to these loci. Mouse mRNAs were obtained from the all_mRNA (mm9) table of the UCSC database. We used only those mouse mRNAs which mapped unambiguously to single loci in the mouse genome. Human and mouse genes at orthologous loci were checked for gene-name identity (using RefSeq gene names from the UCSC Ref Seq RefFlatTable) and non-matching gene names were discarded. Human SAS pairs could retain their SAS structure classification (convergent, divergent, or other) or be structurally different in mouse. In order to assess the incidence of transcription from orthologous loci in which structure is conserved in human and mouse, we used the UCSC Human Genome Browser Assembly hg19 to annotate the loci in human and in mouse.

After our LiftOver genome-wide analysis, we performed a manual annotation of a subset of resulting data (Table 1, row 3 and 4; Supplementary Dataset 5). Gene pairs in which one or both genes were supported only by single, unspliced ESTs without unique sequence anchoring them to the genome were discarded. After this step, human SAS TUs were annotated by obtaining locus coordinates from UCSC. All accession numbers were single-mapping. Using the TU definition, we visually analyzed loci in the UCSC Genome Browser for the following structural features: (1) confirmation of SAS exonic overlap and bidirectional promoters (BDPs) as applicable, (2) the identification and naming of all TUs in the gene pair or the gene chain [defined in Engström et al. (2006)] if present, (3) coding potential for all TUs at the locus. This format was followed identically for annotating conservation of orthologous loci in mouse (UCSC Mouse Genome Browser, mm9 assembly). Structurally non-conserved genes in mouse were identified via BLAT, or reciprocal BLAT: an application of the BLAT interface in which the human gene is matched to the mouse genome and vice versa. Both the human gene and the mouse gene had to be single-mapping and match the other species in terms of orientation and position relative to the nearest orthologous protein-coding genes in order for two transcripts to be deemed positional equivalents (PEs; Engström et al., 2006). Our reciprocal BLAT pipeline entailed obtaining each cDNA sequence using the human Genbank accession numbers as queries in NCBI Entrez. These sequences were used as input for RepeatMasker (http://www.repeatmasker.org) to mask repetitive elements. We used the repeatmasked cDNAs as UCSC BLAT queries and manually annotated the visual browser results.

**Table 1:** **Human-mouse comparative analysis of genomic and transcriptomic orthology at sense-antisense loci**.

**Source**	**Dataset**	**Processing**	**Result**
See “Construction of the human SAS dataset,” Methods	9000 human SAS pair member genes	UCSC LiftOver from Hg19 to Mm9	2227 pairs with genomic orthology
2227 pairs with genomic orthology (above at right)	Lists of genes and pairs with genomic 1:1 orthology in Hg19 and Mm9	Pair non-conservation analysis, see Methods	66 human gene pairs with one gene transcriptionally silent in mouse
Conservation Analysis (above at right)	66 human gene pairs	EST interrogation, see Methods	37 transcriptionally active human gene pairs with one member silent in mouse
EST interrogation (above at right)	37 human gene pairs	Manual annotation of sequence and structure conservation in mouse	N Status
			3 Complete conservation
			7 Positional equivalents
			7 Complete non-conservation
			20 Other

Each row corresponds to a sequential stage in our analysis pipeline. The results (output) from the last column of each row serve as input into the first column of the next row.

### Simple majority rule assignment of mouse SAS pairs to genomic structure types

**Figure 3:**
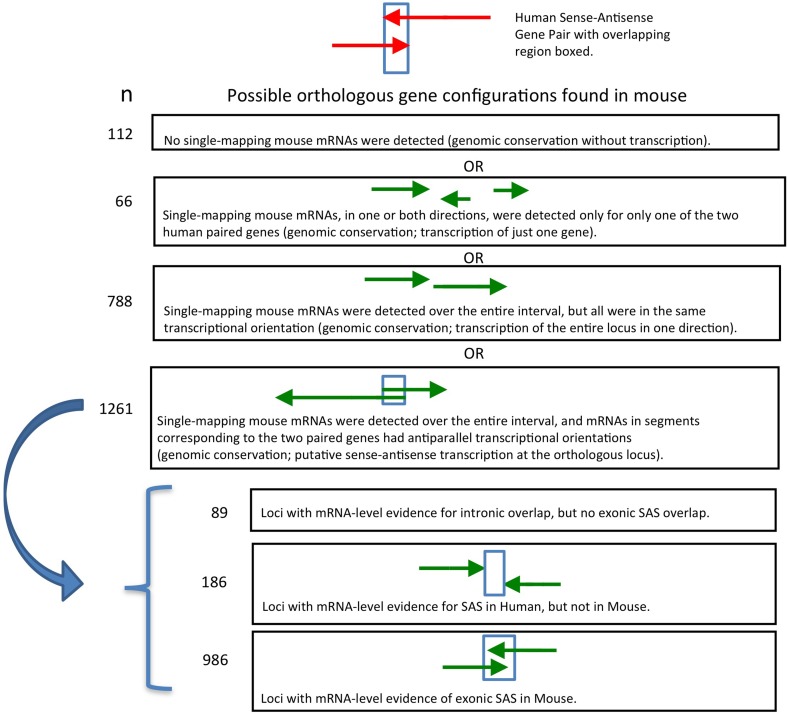
**Genomic structure conservation at 986 SAS pairs putatively orthologous between human and mouse**.

To characterize the genomic structure of mouse orthologs of human SAS genes, we assigned a specific structure to each antisense pair. The structural characterization had to be supported by the majority of mRNAs at each locus; more than half of the mRNAs were required to support the declared structure type. Four structures were possible: convergent, divergent, complex, and no alignment (the first three are shown in Figure 3). Non-aligning pairs, or pairs with ambiguous or “tied” majority-supported structure types, were not considered in downstream analyses requiring this classification.

## Results

### Only 25% of human SAS pairs have both genomic sequence and gene structure conservation in mouse

We began our analysis with 4511 human SAS gene pairs from our prior work. Of our 4511 human SAS pairs 49% (*n* = 2284) lacked unambiguous genomic sequence conservation in mouse, and the other 51% (*n* = 2227, Supplementary Dataset 1, sheet 3) were characterized by single genomic mappings in the mouse for both genes in the same mouse locus. Table 1 summarizes the genomic sequence conservation landscape of human SAS pairs (hg19) along the mouse genome (mm9). The 2227 human/mouse putatively orthologous SAS loci were then assessed for transcription in the mouse.

We analyzed the presence and directionality of transcription at all 2227 mouse putative orthologs of human SAS loci (Supplementary Dataset 2). We found that 1261 of the 2227 mouse loci orthologous to human SAS pairs contain mRNA-level evidence of mouse SAS transcription (Supplementary Dataset 2, sheet 1). In a further 788 orthologous loci in the mouse, only one strand of genomic DNA (plus or minus, but not both) was transcribed, including the entire genomic territory corresponding to the human and mouse SAS locus (Supplementary Dataset 2, sheet 2). We found that in 112 of the 2227 mouse loci, no mouse mRNAs mapped singly and uniquely to either sense-orthologous or antisense-orthologous mouse LiftOver-defined regions of the locus (Supplementary Dataset 2, sheet 3). Finally, in 66 out of 2227 we found that only the sense-orthologous region or the antisense-orthologous region of the mouse locus had matching mRNAs in one or both orientations (Supplementary Dataset 2, sheet 2, “TransAnalysis_OneGenePresent”).

### Genomic sequence conservation of human SAS genes without transcriptional counterparts in mouse: diversity and complexity

We manually annotated the 66 human SAS pairs that had been flagged by our automated pipeline as having genomic sequence conservation for both genes but transcription on either or both strands in the genomic span of only one ortholog in mouse. Our rationale in selecting these loci for annotation was that their human-mouse transcriptional activity difference might be a result of interspecies SAS gene structure distinctions that manual annotation could define at a high resolution. We analyzed these 66 human pairs to determine whether their protein-coding genes had names consistent with a known function (rather than alphanumeric names from large-scale projects), and whether the unnamed genes had protein-coding capacity. Only five pairs were comprised of two functionally named genes in the pair (nine genes were protein-coding and one was an expressed pseudogene), 47 contained one functionally named gene and one non-descriptive alphanumeric identifier, and 14 pairs were comprised of two non-descriptively named (alphanumerically named) genes. Next we examined the longest same-strand open reading frame (ORF) for the 75 genes which had only non-descriptive alpha-numeric identifiers; 24 had ORFs under 100 amino acids (aa) and no BLASTP hits, indicating that they may encode lncRNAs. Further, 17 of those genes had ORFs greater than 100 aa but no conserved domains, putative protein motifs or homology to known proteins. Although these SAS pair members may encode novel non-conserved proteins, the protein-coding potential of these transcripts was not pursued further (Supplementary Dataset 5). To complement our manual annotation, we subjected all input accession numbers to an analysis of protein-coding capacity by the Coding Potential Calculator software (Kong et al., 2007) (Supplementary Dataset 6), and extracted the results into Supplementary Dataset 5 (columns I and J). There was only one case in Supplementary Dataset 5 where a transcript that had not been assigned to a known gene with a descriptive name was categorized by the computational analysis as “coding”: AK128864. The non-protein-coding nature of this transcript is indicated by its genomic position: it is an antisense transcript overlapping the 5′end of the protein-coding gene LCN6 and containing three RepeatMasker repeats in its exons. Therefore, the CPC results generally validate our manual annotation of protein-coding capacity.

We next manually annotated the types of complex loci [SAS pairs and/or gene chains, as defined in Engström et al. (2006) and in Figure 1] represented by the human genes in this set of 66 loci. We discarded 29 human SAS pairs in which full-length transcript evidence was not present (i.e., only EST support from unspliced ESTs, or from ESTs nested on the same strand of known genes, was available; Supplementary Dataset 5, column E). Of these 37 pairs, in human, 21 were standalone SAS pairs, 11 were members of three-gene chains, four pairs belonged to four-gene chains, and one pair belonged to a five-gene chain.

We manually annotated the remaining 37 putative orthologs for gene structure conservation in human and mouse (Supplementary Dataset 5, column M). Gene chain discovery by manual UCSC annotation of the 37 putatively mouse-conserved loci in human resulted in an expansion of the dataset from 74 to 98 TUs. These 98 TUs contained 16 BDPs in human: 12 were at loci with one BDP, and two were at complex loci with two BDPs. In mouse, we analyzed the putatively orthologous loci, based on BLAT of one or both genes, and found a total of 55 TUs, residing at: 13 loci comprised of one transcriptionally active TU and one human to mouse BLAT region of genomic conservation without transcription, 12 loci composed of SAS pairs (including PEs, complete conservation of SAS, and SAS in a different region), and 5 loci containing gene chains. For the remaining 7 human loci, no genomic sequence or gene structure conservation was visible in mouse. These 55 TUs in mouse included those initiating from BDPs at 6 different loci (Table 2).

**Table 2:** **Extent of mouse gene structure conservation for 37 manually annotated human sense-antisense gene pairs**.

**Human, 98 TUs total**		**Mouse, 55 TUs total**	
		No genes at orthologous locus	7
		Single gene at orthologous locus	13
SAS pair	19	SAS pair at orthologous locus	12
3-gene chains	13	3-gene chains at orthologous locus	3
4-gene chains	4	4-gene chains at orthologous locus	1
5-gene chains	1	5-gene chains at orthologous locus	1
No BDPs	23	No BDPs at orthologous locus	31
1 BDP	12	1 BDP at orthologous locus	6
2 BDPs	2	2 BDPs at orthologous locus	0

SAS, sense-antisense; BDP, bidirectional promoter.

**Figure 4:**
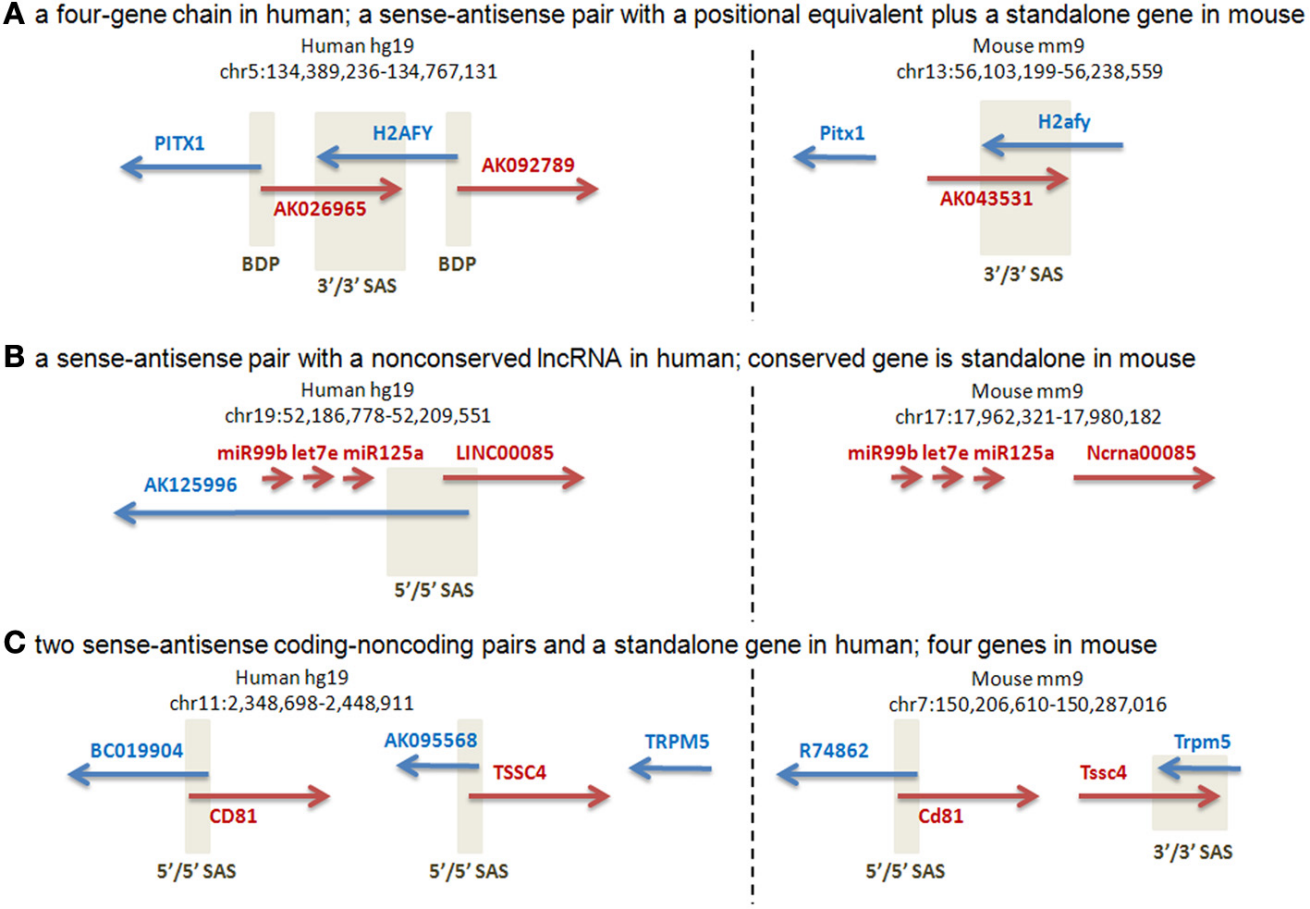
**Manual annotation of selected orthologous loci with human-mouse gene structure distinctions**. Positive-strand transcription, relative to the genome assembly, is in red. Negative-strand transcription, relative to the genome assembly, is in blue. Beige boxes delineate bidirectional promoters (BDP) and sense-antisense overlaps (SAS). A 5′/5′ SAS is an overlap of two genes at their 5′ ends (a divergent overlap). A 3′/3′ SAS is an overlap of two genes at their 3′ ends (a convergent overlap). **(A)** Two protein-coding genes have orthologs: PITX and H2AFY. H2AFY has a positionally equivalent [see Babak et al. (2007) for definition] SAS lncRNA at its 3′end (AK026965 in human) in both species, suggesting a sequence-independent requirement for SAS pairing of H2AFY. In human, H2AFY shares a bidirectional promoter with another lncRNA (AK092789) for which no genomic or transcriptional conservation exists in mouse. (Supplementary Dataset 5: rows 62–63.). **(B)** The human protein-coding gene AY358799 has a mouse ortholog, “Ncrna00085” (encoding a 339-aa protein, despite its misleading name that arose out of incorrect public “lincRNA” annotations that are loaded into the UCSC Genome Database). The same cluster of three conserved microRNAs is observed immediately upstream of this gene in both species. However, this protein-coding gene has a SAS lncRNA, AK125996, only in human. Despite the more comprehensive mouse cDNA/EST coverage by the FANTOM3 data, no antisense cDNAs or ESTs are found at the orthologous mouse locus. (Supplementary Dataset 5: rows 14–15.). **(C)** The human TSSC4 gene overlaps a SAS lncRNA, AK095568, at its 5′ end. The human TSSC4 and TRPM5 genes are clearly separated along the genome, with no intervening transcription. Mouse Tssc4 is SAS to an extended 3′-end isoform of Trpm5, and also lacks any cDNA or EST evidence of a 5′-end SAS transcript. The nearby CD81 gene has a conserved SAS lncRNA in human and mouse. (Supplementary Dataset 5: rows 68–69.).

Our manual annotation of this subset of loci (Supplementary Dataset 5) suggested that the majority (27 of the 37 analyzed) of human SAS pairs were characterized by transcriptional activity differences at the orthologous mouse loci, including different gene structures and absence of human SAS overlaps in the mouse, as judged from all available public full-length cDNA and EST data in Genbank. Three representative cases are shown in Figure 4. The human locus containing the PITX1 (Paired-like homeodomain transcription factor 1) gene is a four-gene chain which joins PITX1, via a bidirectional promoter shared with the lncRNA AK026965, to H2AFY (H2A histone family, member Y isoform), which has a convergent cis-antisense overlap with AK026965 at its 3′end (Figure 4A). H2AFY also has a bidirectional promoter, shared with a second lncRNA, AK092789. There is no mouse ortholog or positional equivalent of AK092789, while AK043531, the mouse positional equivalent of AK026965, is not alignable to AK026965 outside of the H2afy SAS overlap, and does not originate from the mouse Pitx1 promoter. The human PITX1-H2AFY region is a candidate interval for autism, and rearrangements in this region have been linked to Liebenberg Syndrome, a homeotic developmental disorder (Spielmann et al., 2012). The putative regulatory roles of the non-conserved antisense lncRNAs in this region therefore warrant closer scrutiny. The human transcriptional unit AY358799, whose 5′end resides just downstream of a cluster of three microRNAs (miR99B, LET7E, miR125A), has an antisense overlap with the AK125996 transcriptional unit. The overlap resides in the first exons of the two TUs (Figure 4B). AK125996, supported by several independent ESTs, is devoid of ORFs exceeding 100 aa, and is therefore an lncRNA. AY358799, despite corresponding to a public “LINC00085” lncRNA annotation, encodes an ORF of at least 211 aa (source: BC041134) and is therefore a putative protein-coding gene. Its mouse ortholog, “Ncrna00085,” has a 339-aa ORF. The three upstream microRNAs are conserved, but there is no evidence for an antisense transcript, or a bidirectional promoter, in the mouse cDNA and EST data. The human gene TSSC4 (Figure 4C) has a SAS lncRNA, AK095568. Human TSSC4 and TRPM5 are transcriptionally separate. In the mouse, Tssc4 does not have any 5′end SAS transcript comparable to AK095568 in cDNA or EST data, and its promoter is unidirectional. However, mouse Tssc4 and Trpm5 form a SAS overlap; therefore, TSSC4 has different SAS partners in the two species, an lncRNA at its 5′end in human but another protein-coding gene at its 3′end in mouse. Complete gene structure conservation was present in only a minority of the human SAS pairs that were genomically conserved in mouse at the sequence level (*n* = 3), while partial gene structure conservation (*n* = 27) and gene structure non-conservation were more common (*n* = 7).

### The majority of genomically conserved and bidirectionally transcribed human SAS pairs retain their SAS overlap and pair orientation in mouse

Earlier, we found that 1261 of 2227 (56%) human SAS loci had unambiguous mouse orthologs and preliminary evidence of SAS transcription. For each such SAS locus, mRNAs with opposite transcriptional orientation resided within the mouse genomic intervals corresponding to the two individual human paired genes. We proceeded to more precisely interrogate the genomic structure of these 1261 loci. Of these 1261 mouse loci, 186 lacked mRNA-level evidence that gene boundaries of the oppositely oriented transcripts overlapped (Supplementary Dataset 3, sheet 1). An additional 89 loci had mRNA-level evidence for SAS overlaps involving solely introns, but not for exon-exon SAS overlaps (Supplementary Dataset 3, sheet 2). Only 986 mouse orthologs of human SAS loci had mRNA-based evidence of SAS transcription with anti-parallel exon overlaps in mouse (Supplementary Dataset 3, sheet 2).

We analyzed these 986 human and mouse SAS pairs for conservation of pair-structure orientation by comparing mRNA-to-genome alignments at the orthologous loci (Supplementary Dataset 4). In human, these 986 SAS pairs include 250 pairs which are oriented divergently, meaning the exonic SAS overlap occurs at the 5′ end of both genes (Supplementary Dataset 4, sheet 1). Of the 986, 357 human pairs were in the convergent orientation, meaning the exonic SAS overlap occurs at the 3′ end of both genes (Supplementary Dataset 4, sheet 2) and 365 were classified as “other “which incorporates both nested SAS pairs (in which one gene is fully inside another on opposite strands) and further complex orientations (Supplementary Dataset 4, sheet 3). When we annotated the genomic structure of the 986 SAS pairs found at the putative orthologous loci in mouse, we found that merely 43% of gene pair structures are conserved between human and mouse at orthologous loci with evidence of SAS transcription in both species. Human divergent SAS overlaps corresponded to divergent SAS overlaps in 28% of cases, pinpointing this as the least structurally conserved category of human SAS pairs. Human convergent and “other” SAS pairs were equally likely to possess conserved and non-conserved gene pair structures in the mouse.

## Discussion

### Almost half of human SAS gene pairs are genomically non-conserved in mouse

In this work we sought to assess the extent of conservation of SAS pairs between human and mouse, organisms which shared a common ancestor approximately 70 million years ago (Bourque et al., 2004). We examined human SAS gene pairs in mouse at several levels: genomic sequence conservation in publicly available interspecies alignments, gene structure conservation including the presence versus absence of transcriptional activity from each genomic strand of each putatively orthologous locus, and gene pair structure conservation with respect to whether a divergently oriented, convergently oriented, or “other” human SAS pair had an orthologous mouse SAS locus where the orientation of the two overlapping genes recapitulated that in human. In our genome-wide dataset of 4511 human SAS pairs, we found a lack of genomic sequence conservation for one or both genes in the SAS pair (*n* = 2274) in almost half of cases. The existence of SAS gene pairs is a remarkable and non-random phenomenon; it is statistically unlikely for genes to overlap even on a very gene-dense chromosome (Lipovich and King, 2003). Here we reveal the SAS transcriptome to be at the convergence of two unusual events: the greater-than-expected incidence of SAS pairs (Lipovich, 2003) is accompanied by a lack of conservation between two closely related mammals, human and mouse.

We did not further analyze the 1877 human SAS genes which ambiguously mapped to multiple genomic locations in mouse (Supplementary Dataset 1, sheet 1). These sequences might harbor the potential to offer valuable insights into how gene family expansions in rodents, or remaining areas of uncertainty in the mouse genome assembly, relate to the SAS transcriptome. Similarly, we did not pursue the dataset of human SAS pair member genes for which no mouse homologues were identified by LiftOver (Supplementary Dataset 1, sheet 2). These loci merit future work to elucidate their complex evolutionary histories, which may encompass *de novo* gene origination leading to new SAS pair generation along the primate lineage.

### Genomic conservation of human SAS pairs is not a predictor of their transcriptional activity in mouse

We observed that approximately half of human SAS pairs are genomically conserved in mouse, according to the public UCSC-hosted interspecies alignment. Our survey of mouse transcriptome data reveals that only half of those putatively conserved loci give rise to antisense transcription in mouse. Gene structure differences present at orthologous loci despite genomic sequence conservation have been demonstrated for mammalian SAS pairs previously (Veeramachaneni et al., 2004) and can be due to a variety of factors, including but not limited to the creation or destruction of polyadenylation signals after species divergence (Dan et al., 2002), promoter substitutions creating or abolishing transcription factor binding sites, and splice site substitutions after species divergence (Lipovich et al., 2006). Our datasets comprise a versatile resource for the future study of numerous SAS pairs. This resource may yield information about the diversity and prevalence of mechanisms governing the form and function of SAS pairs.

Only a relatively small number of human SAS loci had putative genomic orthologs in the mouse such that no transcription in either direction was observed at those orthologs. In 112 SAS gene pairs, no transcription in the mouse was detected, as no mouse mRNAs mapped uniquely to either sense-orthologous or antisense-orthologous mouse LiftOver-defined regions of the locus. This is consistent with the possibility of gene birth of SAS pairs at loci where there was no transcription in the boreoeutherian ancestor, or alternately gene death of ancient SAS pairs along the primate or rodent lineage, may have taken place (Lipovich et al., 2006). Potential exaptation (Brosius and Gould, 1992) of genomic sequence into SAS spaces may have taken place in the former scenario. Our results suggest that gene structure changes at antisense loci may be prevalent during mammalian evolution and are not limited to isolated case studies. We infer that the conservation of transcriptional status (defined as the presence and orientation of transcription, supported by public cDNA sequences uniquely mapped to each locus under study) does not necessarily follow genomic conservation.

Nevertheless, our assessment of mouse transcriptional activity at genomic orthologs of human SAS loci is tempered by two limitations of our analysis. First, most lncRNAs are expressed at low levels, relative to protein-coding genes (Derrien et al., 2012). This introduces the possibility that a mouse antisense lncRNA might not be represented in any mouse cDNA libraries, while its higher-expressed sense partner would be represented. This limits our ability to detect antisense transcription from full-length transcriptome data, although during our manual annotation of a subset of the data (Supplementary Dataset 5), we considered all mouse evidence for transcription at each locus, including ESTs. Second, as our automated pipeline screened for mouse cDNAs, but not for mouse ESTs, at each mouse ortholog of a corresponding human SAS locus, the pipeline may have missed EST-only evidence for antisense transcription. We adopted a cDNA-only approach to characterizing mouse transcriptional activity in order to more accurately catalog human-mouse differences in SAS pair gene structure (convergent, divergent, or other). Because of the inherently incomplete nature of EST sequences, inferring interspecies pair structure differences using ESTs would have the potential to assign wrong structural classifications to pairs.

### Prevalent chains and BDPs at 66 human SAS gene pairs showcase mouse gene structure differences

We identified 66 human SAS gene pairs which had conservation of the entire pair interval at a single genomic location in the mouse but detectable transcriptional activity for only one of the two expected mouse orthologs. We hypothesized that these human pairs would be particularly illustrative of human-mouse SAS pair gene structure differences because they potentially contained entire genes transcribed in human but not in mouse, pointing to gene birth in conserved sequence or other complexity. To characterize the gene structure conservation of these 66 human SAS pairs, we performed UCSC Genome Browser manual annotation and characterized the extent of SAS, BDPs, gene chains, and lncRNAs at each locus, along with gene structure conservation in the orthologous mouse locus. We found that most of the human genes in this analysis had only non-descriptive alphanumeric names assigned by high-throughput transcriptome projects in the databases, indicating that they encode previously uncharacterized proteins or lncRNAs (Jia et al., 2010). The lack of named known genes implicates TUs at these loci as a reservoir of new functions, a property which has been suggested to characterize lncRNAs (Guenzl and Barlow, 2012). However, unbiased proteogenomic mapping (Bánfai et al., 2012) would be necessary to formally exclude the possibility that these cDNA-supported TUs at complex non-conserved SAS loci might be translated into proteins.

To further compare gene structure conservation between human and mouse SAS pairs, we manually annotated 37 high quality human SAS pairs (composed of transcripts which retained cDNA support, not just EST support, in hg19) from the dataset of 66 above. Surprisingly, we found that half of these pairs were parts of longer complex loci, gene chains (Engström et al., 2006): 18 loci which were composed of between three and five genes. In mouse, at the 30 loci orthologous to these human genes, we found only five loci composed of more than just the two genes in a SAS pair. This result is noteworthy when the sources, and the numbers, of public cDNA sequences are considered in the human and mouse. Thanks to the FANTOM3 project (Katayama et al., 2005; Carninci and Hayashizaki, 2007), the mouse has an unparalleled collection of approximately 600,000 cDNA clones corresponding to over 250 tissues and cell types. Human cDNA and EST sequencing efforts have not been nearly as comprehensive. Yet, despite the greater depth of mouse, relative to human, transcriptome coverage in public cDNA data, our SAS gene pair analysis indicates reduced complexity (pairs, instead of chains), and reduced, rather than increased, gene counts at the mouse orthologs of these human gene chains. The examples in Figure 4 are representative of this difference. However, more comprehensive assessments of human-mouse gene structure differences at complex loci will only become possible with increased coverage of additional tissues and cell types by full-length cDNA clones and RNAseq-derived transcript models. The utility of currently available second-generation RNAseq datasets is limited by a lack of the full-length transcript models that would be necessary for the derivation and interspecies comparison of gene structures at complex loci.

The most common situation in our cross-species analysis was a human locus, composed of a single SAS pair or containing a SAS pair as part of a gene chain, where one or both genes in the mouse locus orthologous to that SAS pair lacked any evidence of transcriptional activity in Genbank cDNAs (*n* = 27). In these situations, mouse loci orthologous to human SAS pairs contained only one gene, as a possible result of either a gene birth on the primate or a gene loss on the rodent lineage. Positional equivalency [the presence of SAS pairing at an orthologous locus in two species, where one member of the pair is a conserved protein-coding gene while the other lacks any sequence conservation outside of the actual SAS overlap (Engström et al., 2006)] was found in almost 25% of cases (*n* = 7). Interestingly, three of these seven loci encode known protein-coding genes whose products have DNA-binding domains, suggesting that complex loci containing positional equivalents may impact transcriptional regulation in both human and mouse. Manual annotation using the UCSC Genome Browser for visual interrogation of complex loci was integral to this analysis. Even though our manual annotation focused on a minority of pairs, these 66 pairs represent a microcosm of the evolutionary complexity of the mammalian SAS transcriptome.

**Figure 5:**
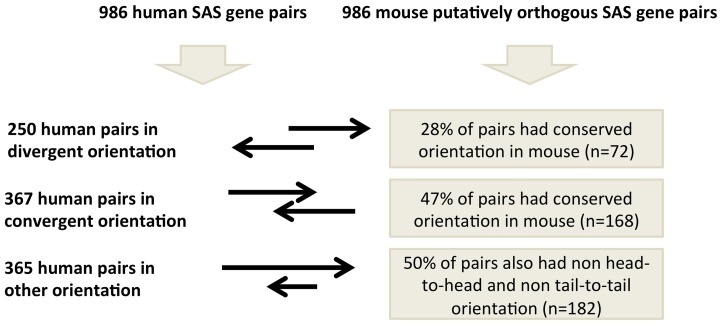
**Transcriptional activity at the 2227 mouse orthologs of human sense-antisense loci**.

Complete SAS gene structure conservation was found in only three cases suggesting that in these cases purifying selection may have affected SAS gene structures, not solely gene sequences, between human and mouse. The protein-coding genes in these cases encoded a chaperone that interacts with epigenetic remodeling factors (DNAJB8), a protein associated with cell division as well as with chromatin states in the interphase nucleus (NUMA1), and a tumor suppressor originally discovered from a B-cell lymphoma translocation breakpoint (BCL7A). However, the most common category of human-mouse gene structure relationships was non-conservation (Figure 5). This result was not expected, given the high sequence similarity between human and mouse genomes and the fact that the structure of protein-coding genes is generally similar between mammalian orthologs.

### Certain human SAS pairs are genomically conserved and bidirectionally transcribed in mouse

We identified only 986 mouse orthologs of 4511 human SAS loci such that both the genomic sequence of the two genes in each SAS pair and their bidirectional transcription, including the SAS overlap itself, were conserved in mouse (Figure 5). Because this is a minority of the human SAS pairs, transcriptional activity at mouse orthologs of human SAS loci is more frequently characterized by interspecies differences in gene structure, some of which exist despite genomic sequence conservation. Having considered the conservation of both genomic sequence and the presence as well as the directionality of transcription at orthologous SAS loci, we investigated an additional SAS pair property whose conservation can be tested: the genomic structure category to which each SAS pair belongs (Figure 3). To investigate the extent of gene structure conservation at genomically and transcriptionally conserved SAS pairs, we analyzed each of those 986 human and orthologous mouse SAS loci for the genomic structure category of the pair: convergent, divergent, or other. Summarily, 43% of conserved and bidirectionally transcribed SAS gene pairs in human and mouse belonged to the same genomic structure category in both species, suggesting that constraints on the genomic structure may be relaxed, despite the joint conservation of sequence and bidirectional transcriptional activity.

When we compared genomic structure categories between complete SAS orthologs, we found that when the human pair was divergent (i.e., when the 5′ ends of the sense and antisense genes overlapped), its orthologous pair was most often in the complex, “other,” category (48%) in the mouse. This is unexpected because, if genomic structure were to be as consistently conserved as sequence and transcriptional activity in this subset of pairs, then we would expect the orthologs of human divergent pairs to be divergent in mouse as well. In fact, none of the three SAS genomic structure categories was found in more than half of the mouse orthologs of the human SAS pairs that had been assigned to that category (Figure 3). Aside from evolutionary lineage-specific genomic structure differences at conserved loci, an alternate explanation is that the availability of more full-length cDNAs in the mouse than in human, as a result of the FANTOM Consortium and in contrast to the more frequently 5′-truncated nature of human cDNAs, might systematically alter genomic structure classifications in mouse.

SAS pairs reflect considerable and non-conserved gene structure complexity, which is particularly interesting as a source of interspecies regulatory differences at the 3′ends of protein-coding SAS pair member genes: antisense lncRNA binding to a sense mRNA 3′UTR may obstruct a miRNA binding site in that UTR, potentially protecting the mRNA from miRNA-induced post-transcriptional suppression. This process, by which the 3′ends of SAS pair member genes' mRNAs may compete with cognate lncRNAs, and/or with trans-encoded mRNAs, for miRNA binding has been characterized as “competing endogenous RNA” regulation (Salmena et al., 2011). Our results suggest that protein-coding transcripts from orthologous loci may vary in their usage of this regulatory mechanism, because gene structure at the 3′ ends of the protein-coding genes, and the presence as well as the extent of antisense transcripts overlapping those 3′ ends, vary from species to species. The 3′ ends of genes perform a wide repertoire of regulatory roles, including the initiation of antisense transcription (Murray et al., 2012). We observed that when the human pair overlap was convergent, the mouse pair also most often fit this category (47%). Future work in this field should address whether selective pressures driven by the miRNA/antisense lncRNA competition are a reason for the relatively frequent conservation of convergent SAS overlaps.

### Our datasets identify candidate loci with non-conserved, terminal-end-dependent regulation

Among SAS gene pair structural category types in human and mouse (divergent, convergent, or “other”), 42% of pairs display the same pair structure. The lack of pair structure conservation in the remaining group hints at a diversity of regulatory mechanisms through which lncRNAs at orthologous loci may exert regulation, and be regulated, in different evolutionary lineages. We also identified SAS pairs with both genomic conservation and transcriptional activity conservation, but with an apparently flipped region of exonic SAS overlap: for example, a SAS pair is divergent in human, but its putative ortholog is convergent in mouse. These SAS pairs may harbor conserved protein-coding genes overlapped by non-conserved terminal-end-overlapping antisense lncRNAs, pointing to different antisense mechanisms of regulating an orthologous protein-coding gene in different species. We provide a resource that can be used to identify non-conserved terminal-end-overlapping antisense transcripts (Supplementary Dataset 4).

In bacteria, riboswitches change the 5′end structure of mRNAs in response to specific conditions, altering translational competence. Riboswitch sequences are contained within these mRNAs (Breaker, 2012). Widespread occurrence of antisense transcription suggests a conceptually similar model in which an antisense transcript reversibly remodels a sense mRNA in eukaryotes, by changing the availability of the overlapping mRNA region for protein interactions or post-transcriptional modifications. The promiscuous nature of certain dsRNA binding proteins, which lack sequence specificity or interact with a wide assortment of degenerate RNA motifs, means that existing RNA-binding protein infrastructure is capable of interacting with non-conserved, newly arisen antisense lncRNAs.

Different regulatory modalities may be used by antisense lncRNAs based on nuclear versus cytoplasmic localization. It is not clear whether cytoplasmic lncRNAs possess sufficient stability or half-life to harbor regulatory potential. Evidence is emerging for a large repertoire of lncRNA mechanisms, some of which are nuclear and some cytoplasmic. Long RNAseq data being generated by the ENCODE Consortium enables transcript localization and nuclear to cytoplasmic ratio approximation in any profiled cell type, providing a first step toward distinguishing lncRNA, including antisense, mechanisms based on subcellular localization. In this study, we have focused on SAS gene pairs with shared sense and antisense exonic sequence. These pairs may exert their regulatory potential through both epigenetic and cytoplasmic mechanisms, whereas intronic SAS overlaps are more suggestive of nuclear function, since all pre-mRNAs prior to splicing in the nucleus contain these sequences while relatively few retained introns are observed in transcripts exported to the cytoplasm. The same antisense transcript might act through different mechanisms in different tissues and time points.

### Recent evolution places sense-antisense pairs at the interface of RNA structure and function

Genomic regions with non-conserved sequences between human and mouse display higher potential for secondary structure conservation of their encoded RNAs than what would be expected by chance (Torarinsson et al., 2006). These non-conserved regions with conserved secondary structure may encode structural lncRNAs bearing similar functions based not on sequence identity but on genomic structure, including the presence of antisense transcription itself. In this study, we manually explore the level of both genomic sequence and gene structure conservation of a subset of non-conserved loci and find a high proportion of antisense lncRNAs in complex loci with other genes. These gene chains raise the possibility that non-conserved secondary structures are associated with cis-regulation.

In 1975 King and Wilson (King and Wilson, 1975) suggested that distinct regulation of protein-coding genes in closely related species is caused by sequence differences in non-protein-coding DNA, noting that human and chimpanzee proteins are nearly identical despite the pronounced interspecies phenotypic distinctions. Consistent with emerging evidence for multiple regulatory roles of lncRNAs, our results indicate that interspecies differences in the regulation of protein-coding genes may be encoded by non-conserved antisense lncRNAs. We have posited that *de novo* genes at SAS loci in human may have arisen through gene birth including co-option of transposable elements or other processes (Lipovich, 2003). We infer that human SAS loci may be enriched for primate-specific regulatory functions. The thousands of lncRNAs that are simultaneously primate-specific and brain-expressed (Derrien et al., 2012) should be systematically interrogated for evidence of such roles, which would bring us closer to understanding the relationship between lncRNA and the overarching question of what makes humans human. The lack of full-length cDNA sequences and assembled RNAseq transcript models in current non-human-primate transcriptome data still hinders accurate analysis of complex loci in primates.

SAS gene pairs may arise as a consequence of retroposition (Zhu et al., 2009), while de novo birth of genes with regulatory functions can take place at non-coding and formerly non-genic DNA regions (Carvunis et al., 2012). However, these observations have not been previously placed within the framework of global gene structure studies of the human SAS transcriptome. Our results provide a foundation for future studies in this area through a survey of sequence, structure, and transcriptional activity conservation of the human SAS transcriptome.

An early study of well-annotated protein-coding genes showed that less than half of human SAS pairs had mouse homologs for both genes in the pair, and that over half of the latter were not conserved structurally, as mouse lacked a SAS overlap of the homologous genes (Veeramachaneni et al., 2004). Our findings extend upon those nearly decade-old results through the use of a far larger dataset with additional conservation metrics enhanced by manual annotation. We have analyzed an order of magnitude more loci than that earlier study, and we have provided fine-resolution mapping of gene structure differences between orthologs, rather than merely testing for the presence of orthologous SAS overlaps. Our results canvass the broad coexistence of mRNA conservation and lncRNA non-conservation, at both gene sequence and gene structure levels, in the SAS transcriptome, along with a central role for antisense lncRNAs as linchpins of the interspecies distinctions of both gene structure and transcriptional activity at SAS loci.

Numerous lncRNAs regulate protein-coding target genes, both in-cis and in-trans, though epigenetic mechanisms. Thousands of mammalian lncRNAs have been documented by a high-throughput RIPseq strategy to bind the PRC2 complex (Lee, 2012), and these interactions are increasingly realized to be contributors to human disease (Lipovich et al., 2012; Modarresi et al., 2012). We reveal a universe of nearly 5000 SAS loci, the majority of which contain lncRNAs. Through the potential of these antisense lncRNAs to regulate their SAS pair partner protein-coding genes, these loci signify potential opportunities for therapeutic targeting of antisense-mediated gene regulation.

### Conflict of interest statement

The authors declare that the research was conducted in the absence of any commercial or financial relationships that could be construed as a potential conflict of interest.
